# Exploring the Metabolic Effects of a Herbal Remedy of *Asarum sieboldii*, *Platycodon grandiflorum*, and *Cinnamomum cassia* Extracts: Unraveling Its Therapeutic Potential as a Topical Application for Atopic Dermatitis Treatment

**DOI:** 10.3390/antiox13050563

**Published:** 2024-05-02

**Authors:** Gakyung Lee, Byung Hwa Jung, Taemin Lee, Jae Hyeon Park, Hyung Sik Kim, Hocheol Kim, Hyun Ok Yang

**Affiliations:** 1 Department of Integrative Biological Sciences and Industry, Sejong University, Seoul 05006, Republic of Korea; lgg1025@sejong.ac.kr (G.L.); alsdl5454@naver.com (T.L.); 2 Convergence Research Center for Natural Products, Sejong University, Seoul 05006, Republic of Korea; 3 Center for Advanced Biomolecular Recognition, Korea Institute of Science and Technology (KIST), Seoul 02792, Republic of Korea; jbhluck@kist.re.kr; 4 Division of Bio-Medical Science and Technology, KIST School, University of Science and Technology, Seoul 02792, Republic of Korea; 5 School of Pharmacy, Sungkyunkwan University, 2066, Seobu-ro, Jangan-gu, Suwon 16419, Republic of Korea; sky3640@skku.edu (J.H.P.); hkims@skku.edu (H.S.K.); 6 Department of Herbal Pharmacology, College of Korean Medicine, Kyung Hee University, 26 Kyungheedae-ro, Dongdaemun-gu, Seoul 02447, Republic of Korea; hckim@khu.ac.kr

**Keywords:** atopic dermatitis, metabolomics, topical agent, inflammation

## Abstract

Our previous study demonstrated that our novel herbal remedy, a mixture of *Asarum sieboldii*, *Platycodon grandiflorum*, and *Cinnamomum Cassia* extracts, exhibits a therapeutic effect in 1-chloro-2,4-dinitrobenzene (DNCB)-induced mice by inhibiting the Th-2 inflammatory response upon oral administration. It also ameliorated imbalances in lipid metabolism related to the skin barrier function in keratinocytes, indicating its potential as a topical agent. This study aims to further investigate the therapeutic effects and metabolic mechanisms of its topical application. The anti-atopic effect was evaluated using dermatitis scores, histopathological analysis, and immune cell factors in DNCB-induced mice. Metabolomic profiling of serum and lesional skin was conducted to elucidate the metabolic mechanisms. The topical application significantly reduced dermatitis scores, mast cell infiltration, and serum levels of immunoglobulin E (IgE), IFN-γ, interleukin (IL)-4, IL-17, and thymic stromal lymphopoietin (TSLP), demonstrating its effectiveness in treating atopic dermatitis (AD). Serum metabolomics revealed alterations in fatty acid metabolism related to the pro-inflammatory response. In lesional skin, metabolic markers associated with oxidative stress, immune regulation, and AD symptoms were restored. This study demonstrated its potential as a topical agent in suppressing Th-2 inflammatory responses and improving metabolic abnormalities related to AD symptoms, providing crucial insights for developing natural AD treatments.

## 1. Introduction

Atopic dermatitis (AD) is a chronic relapsing inflammatory skin disorder characterized by immune system imbalance and dysfunction of the epidermal skin barrier [1]. Various symptoms, including itching, dryness, eczema, and cracking, lead to physical and mental stress and sleep disturbances and severely interfere with the patient’s quality of life [2]. Therefore, controlling AD symptoms, along with the treatment of immune and skin barrier dysfunction, is an important adjuvant strategy for optimized long-term management [3]. Several treatments are available for AD, including emollients, glucocorticosteroids, and immunosuppressants. Nevertheless, long-term use may lead to significant side effects, such as skin thinning [4]. Therefore, alternative treatments for AD using natural products that are safe for long-term use with low toxicity and few side effects have been studied [5].

BS012 is a novel herbal remedy consisting of *Asarum sieboldii*, *Cinnamomum cassia*, *and Platycodon grandiflorum* extracts, identified using a high-throughput bioassay from Socheongryong-tang, a traditional herbal decoction used to treat allergic rhinitis [6]. In our previous studies, the reported synergistic inhibitory effect of BS012 on T-helper 2 (Th2) cell differentiation [7] prompted investigation of its anti-inflammatory potential both in vitro and through oral administration in an in vivo model of AD [8]. These findings revealed a significant improvement in the pathological state of AD, including enhanced systemic anti-inflammatory metabolism, following the oral administration of BS012. In Tumor necrosis factor-alpha/interferon-gamma (TNF-α/IFN-γ)-stimulated keratinocytes, BS012 restored the lipid metabolic imbalance, contributing to improved skin barrier function. Therefore, we propose BS012 as a promising topical agent.

In the context of herbal extracts, elucidating the underlying mechanism is challenging because of the complex interactions between the activities of independent components within their diverse compositions. Metabolomics has emerged as a powerful “system-to-system” approach for understanding therapeutic mechanisms by providing comprehensive characteristics related to physiological and pathological conditions in biological systems [9].

The primary objective of this study was to evaluate the therapeutic effect of BS012 as a topical agent for AD treatment in a 1-chloro-2,4-dinitrobenzene (DNCB)-induced mouse model. The secondary aim was to elucidate the underlying metabolic mechanisms responsible for these therapeutic effects. The complex interplay between these mechanisms within the whole body and lesional skin has been identified using serum and skin tissue metabolomics strategies. Therefore, we aimed to evaluate the potential of BS012 as a topical agent for treating AD based on insights from AD-related markers and metabolic mechanisms.

## 2. Materials and Methods

### 2.1. BS012 Extract Preparation

BS012 was prepared by combining the extracts of *A. sieboldii*, *C. cassia*, and *P. grandiflorum.* Samples were acquired from the Kyungdong Market (Woori Herb), Seoul, Republic of Korea, in January 2016. The materials were verified by one of the authors (H. K). The dried samples were grounded and extracted with 70% ethanol (concentration of 0.1 kg/L) twice, each for a duration of 3 h at room temperature. Subsequently, the obtained extracts were condensed at a reduced pressure within the temperature between 60 °C and 70 °C. For the BS012 formulation, the proportions of each freeze-dried extract, *A. sieboldii*, *C. cassia*, and *P. grandiflorum*, were weighed and mixed in a specified ratio of 1:2:2. The herbal powder was stored at −20 °C until use. The composition of BS012 used in this study was the same as that used in the previous study, and the composition profiling was reported in the previous study [8].

### 2.2. Animal Housing

Six-week-old male NC/Nga mice were obtained from Central Laboratory Animal Inc. (Seoul, Republic of Korea). The mice were kept in a controlled environment with pathogen-free conditions, including 50  ±  15% humidity, 23  ±  2 °C, and a light–dark cycle of 12 h. The research protocol received approval from the Sungkyunkwan University Laboratory Animal Care Service (SKKUIACUC2022-05-18-1).

### 2.3. DNCB Induction of AD-Like Skin Lesions 

**Figure 1:**
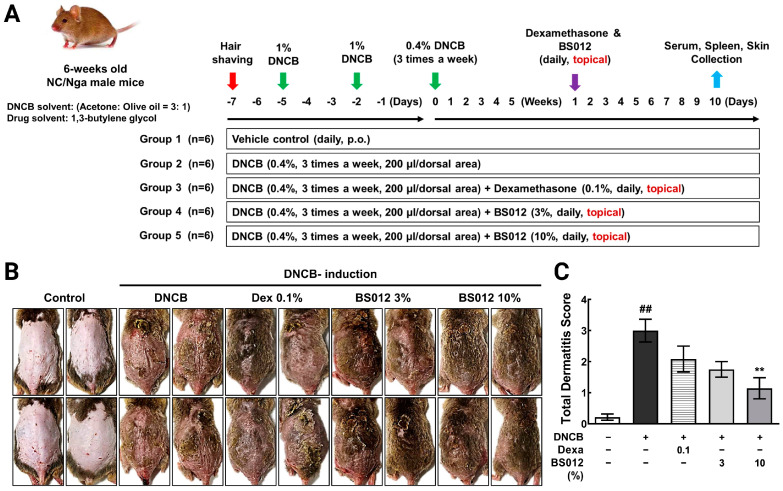
Impact of BS012 on symptoms resembling atopic dermatitis (AD) induced by DNCB in mice. (**A**) Outline of the animal experimental design. (**B**) Representative images of the dorsal skin from four representative mice per group after treatment, based on the total dermatitis score. (**C**) Comprehensive dermatitis score. Data in the graphs are expressed as mean ± SEM for *n* = 6; ## *p* < 0.01 vs. control group; ** *p* < 0.01 vs. DNCB-induced group; Dexa: Dexamethasone.

AD-like skin lesions were established in NC/Nga mice (male, six-week-old) using DNCB, following the method described by Fujii et al. [10]. Figure 1A shows the animal experiment scheme, including tissue collection, to assess the in vivo anti-atopic efficacy mechanism of BS012 through topical application. Following a week of acclimatization, the mice were randomly divided into five groups (*n* = 6 per group). These groups included the non-treated group (control), DNCB-induced group (negative control), a group treated with DNCB and 0.1% dexamethasone (positive control), and groups treated with DNCB and BS012 (3% or 10%). Prior to the experiment, the dorsal hair was trimmed using an electric clipper, followed by the application of a depilatory cream to eliminate any leftover hair. A 200 μL solution of 1% DNCB was prepared by dissolving it in a mixture of acetone and olive oil (3:1). To induce AD-like lesions, this solution was applied topically to the dorsal skin of the mice on the first and fourth days using a brush. After 7 days, a solution containing 0.4% DNCB was applied to the dorsal skin similarly. This application was repeated three times a week for 5 weeks. BS012 (3% or 10% *w*/*v*) and dexamethasone (0.1% *w*/*v*) in 1,3-butylene glycol was topically applied to the skin once a day for 10 consecutive days (Figure 1A). Mice were sacrificed after 24 h following the final treatment.

### 2.4. Quantification of the Total Dermatitis Score

The total dermatitis scores were assessed 24 h after the last treatment, applying slightly adjusted criteria as described by Lim et al. [11]. The skin severity score, which could reach a maximum of 15, was determined by adding the individual scores for five specific signs and symptoms: edema, erythema/hemorrhage, excoriation/erosion, lichenification, and scaling/dryness. The scores were assigned ranging from 0 (no symptoms) to 3 (severe symptoms). Clinical symptoms were quantified using images taken with an MV800 digital camera (Samsung Inc., Seoul, Republic of Korea).

### 2.5. Assessment of Serum Immunoglobulin (IgE) and Inflammatory Cytokine Levels

Abdominal aorta blood samples were obtained and preserved at −80 °C until they were used. The blood was centrifuged at 3000× *g* for 15 min at 4 °C to obtain serum samples. The concentrations of IgE, interleukin (IL)-6, IL-17, IFN-γ, and thymic stromal lymphopoietin (TSLP) in the serum were determined using ELISA kits (Abcam, Cambridge, UK). The serum concentration of IL-4 was quantified using ELISA kits (R&D Systems, Minneapolis, MN, USA).

### 2.6. Histological Analysis

To assess the therapeutic efficacy of BS012, histological alterations were quantified 24 h following the final treatment. The dorsal skin was immobilized in 10% neutral buffered formalin, encased in paraffin, and separated into 4 μm thick sections. The epidermal thickness was assessed using hematoxylin and eosin (H&E) staining. Toluidine blue was used to stain mast cells in the skin. Mast cells were counted in 10 randomly selected regions for each mouse using a BX51 microscope from Olympus, Tokyo, Japan, set at a magnification of 100×. The evaluations were conducted blind in which the participants were unaware of certain information.

### 2.7. Scratching Behavior

To examine alterations in scratching behavior in NC/Nga mice, we quantified the frequency of scratching of the neck, nose, ears, and dorsal skin for 15 min. The total number of scratching behaviors was calculated as the mean of the individual assessments.

### 2.8. Sample Preparation and MS-Based Metabolomic and Lipidomic Profiling 

Serum samples were prepared using a simple protein precipitation procedure, as previously described [12]. Lesional skin tissue was finely chopped and stored at −80 °C. A total of 300 mg of tissue was homogenized in 900 μL of 70% MeOH and then centrifuged for 10 min, and 500 μL of the supernatant was transferred into a new tube. The supernatant was evaporated and reconstituted with 50 μL of 70% MeOH. The final diluted samples were transferred to LC vials and analyzed using a LTQ Orbitrap Velos Pro mass spectrometer (MS) connected to an Ultimate 3000 UHPLC system (Thermo Scientific, San Jose, CA, USA). The instrumental analysis conditions were consistent with those of a previous study [12]. Raw MS data were preprocessed using Xcalibur 2.2 and Compound Discoverer 3.3 software (Thermo Fisher Scientific, Waltham, MA, USA). Multivariate analysis was conducted using SIMCA 17 software (Umetrics, Inc., Ume, Sweden) and MetaboAnalyst (http://www.metaboanalyst.ca/ (accessed on 22 December 2023)). The Wilcoxon rank-sum test was conducted using R studio (https://www.r-project.org/ (accessed on 26 October 2023)) to evaluate the statistical significance between the control and DNCB-induced groups, as well as between the DNCB-induced and BS012-treated groups. Metabolites that showed significant changes were identified as previously described [12]. The compounds derived from BS012 in the skin tissue were profiled to investigate the effect of BS012 components absorbed into the skin. These compounds were identified as described above.

## 3. Results

### 3.1. Effect of Topically Applied BS012 on DNCB-Induced Atopic Skin Lesions

**Figure 2:**
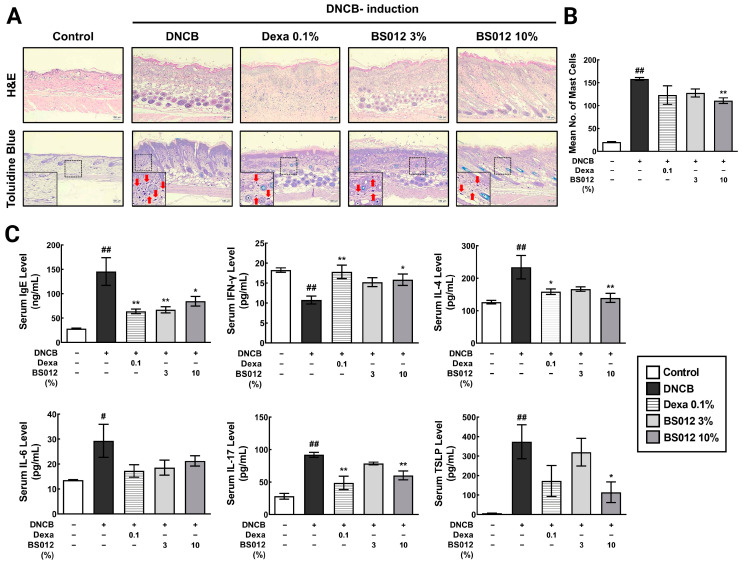
Influence of BS012 on clinical atopic inflammation severity induced by DNCB. (**A**) Histological examinations using H&E and toluidine blue staining at a magnification of 100×. The solid square is an enlarged section of the square outlined by the dotted line, with mast cells highlighted by red arrows. (**B**) Assessment of mast cell infiltration in 10 randomly selected regions for each mouse skin using toluidine blue staining. (**C**) Serum levels of cytokines and chemokines. Data in the graphs are expressed as mean ± SEM for *n* = 6; # *p* < 0.05, ## *p* < 0.01 vs. control group; * *p* < 0.05, ** *p* < 0.01 vs. DNCB-induced group; Dexa: Dexamethasone.

To examine the impact of BS012 on the AD mouse model, we induced dorsal skin inflammation in NC/Nga mice using DNCB (Figure 1B). The topical administration of DNCB resulted in atopic dermatitis-like lesions characterized by edematous erythema, dryness, and scratches. The efficacy of DNCB was compared to that of dexamethasone, a medication used to treat immune-mediated skin diseases. Spleen weight was measured to assess the effect of the topical application of BS012 on systemic immune responses. BS012 treatment decreased spleen weight compared with the enlarged spleen resulting from DNCB treatment (Figure S1). Subsequently, we assessed clinical symptoms, including scratching behavior and a total dermatitis score. After AD induction, the scratching count significantly increased in DNCB-treated NC/Nga mice compared to that in normal mice on day 1 post-treatment (Figure S2). However, the scratch count was reduced in mice treated with 3% or 10% BS012 or dexamethasone on day 10 post-treatment, which counteracted the DNCB-induced increase in the scratch count. The total dermatitis score was substantially higher in AD-induced mice than that in the control group (Figure 1C). The severity of the condition was reduced in both the dexamethasone and BS012 treatment groups. However, a significant reduction was observed in the high-concentration (10%) treatment group with BS012. Histological examination of the dorsal skin from the untreated group of mice stained with H&E or toluidine blue revealed normal tissue characteristics. Mice in the DNCB group displayed epidermal hyperplasia, and infiltration into mast cells (Figure 2A and Figure S3). Compared to control mice, DNCB-treated mice had a thicker epidermis. Applying BS012 topically improved the keratinized layer depth and provided mast cell count to levels similar to those seen in the dexamethasone group (Figure 2B). The high-resolution original images of histological analysis are shown in Figure S4.

### 3.2. Impact of BS012 on Serum Cytokines and Chemokine Levels in DNCB-Induced NC/Nga Mice 

The levels of IgE, IL-4, IL-6, IL-17, and TSLP were significantly higher in the DNCB group than those in the control group. Treatment with BS012 led to a decrease in these cytokines and chemokines, demonstrating an immunomodulatory effect. In addition, the administration of BS012 resulted in an elevation of serum IFN-γ levels, which were decreased in response to the development of atopic skin lesions (Figure 2C). This result showed more significant changes in IL-17 and serum IFN-γ than with the oral administration of BS012 and indicates that more diverse inflammatory markers in serum were changed by topical application.

### 3.3. Changes in Serum Metabolites following Topical Application of BS012 in DNCB-Induced NC/Nga Mice

**Figure 3:**
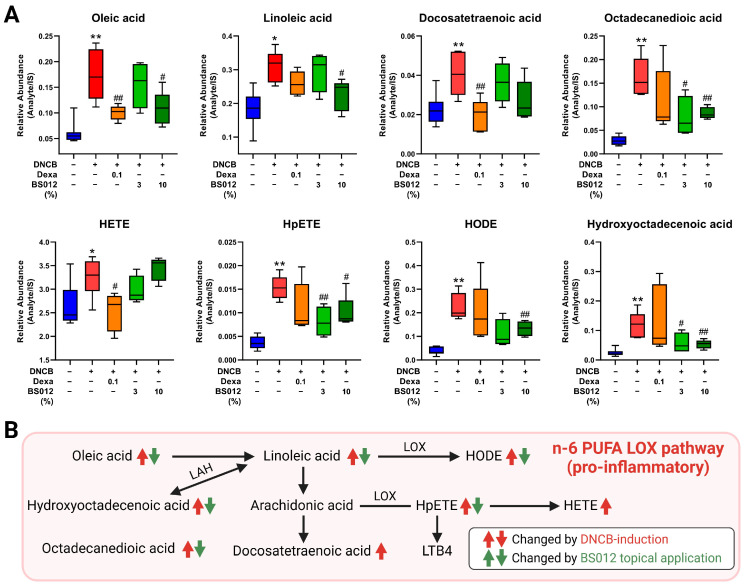
Effects of BS012 topical application on serum metabolites in mice induced with DNCB. (**A**) The significant alterations in the levels of identified metabolites associated with fatty acid metabolism in the serum. (**B**) Changes in metabolic pathways related to n-6 poly unsaturated fatty acids (PUFAs) and lipid mediator. Graphs represented as mean ± SEM, *n* = 6; * *p* < 0.05, ** *p* < 0.01 vs. Control group; # *p* < 0.05, ## *p* < 0.01 vs. DNCB-induced group; Dexa: Dexamethasone.

Serum metabolomic analysis was performed to explore the systemic changes induced by DNCB and BS012. The serum metabolomic profiles of each experimental group exhibited distinct clustering. We identified 16 metabolites that exhibited significant differences between the control and DNCB-induced groups, which were restored in the dexamethasone and BS012 groups (Figure S4). Most identified metabolites were associated with lipid metabolism, especially fatty acid (FA) metabolism (Table S1). This metabolic shift is linked to the systemic inflammation triggered by DNCB. A significant increase in several FAs was observed, reversed by treatment with either dexamethasone or BS012. Many of the metabolites affected were related to n-6 polyunsaturated fatty acids (PUFAs) and lipid mediators (Figure 3A). The levels of n-6 PUFAs, such as linoleic acid and docosatetraenoic acid, and related lipid mediators, including hydroxyoctadecadienoic acid (HODE), hydroxyeicosatetraenoic acid (HETE), and hydroperoxyeicosatetraenoic acid (HpETE), were significantly upregulated following DNCB induction. These changes exhibited a close correlation with n-6 PUFAs and the lipid mediators derived from them via the Lipoxygenase (LOX) pathway. This metabolic pathway was upregulated following AD induction and downregulated by the application of BS012 (Figure 3B). The BS012 treatment group showed downregulation of these metabolites, suggesting a potential recovery effect of BS012 on the induced metabolic perturbations. 

### 3.4. Changes in Skin Lesion Metabolites following the Topical Application of BS012 in DNCB-Induced NC/Nga Mice

**Figure 4:**
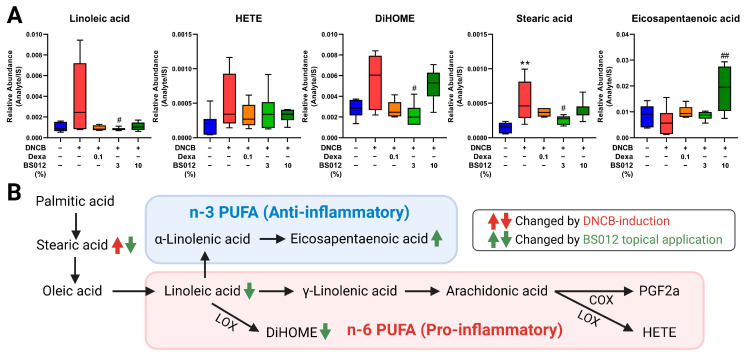
Effect of topical application of BS012 on fatty acid (FA) metabolism in skin tissue of DNCB-induced mice. (**A**) The changed levels of identified metabolites associated with FA metabolism. (**B**) Changes in metabolic pathways related to n-3 poly unsaturated fatty acids (PUFAs), n-6 PUFAs, and lipid mediator. Graphs depict mean ± SEM for *n* = 6; ** *p* < 0.01 vs. control group; # *p* < 0.05, ## *p* < 0.01 vs. DNCB group; Dexa: Dexamethasone.

**Figure 5:**
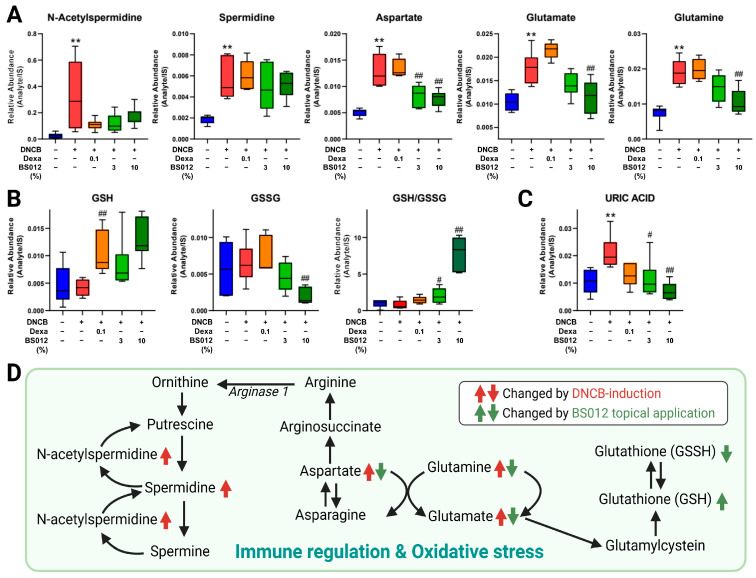
Effect of topical application of BS012 on skin tissue metabolites related to immune and oxidative stress regulation in DNCB-induced mice. The changed levels of identified metabolites related to (**A**) polyamine, glutamine, (**B**) glutathione (GSH), (**C**) uric acid metabolism showed significant changes in skin tissue. (**D**) Changes in metabolic pathways related to polyamine, glutamine, and GSH. Graphs depict mean ± SEM for *n* = 6; ** *p* < 0.01 vs. control group; # *p* < 0.05, ## *p* < 0.01 vs. DNCB group; Dexa: Dexamethasone.

**Figure 6:**
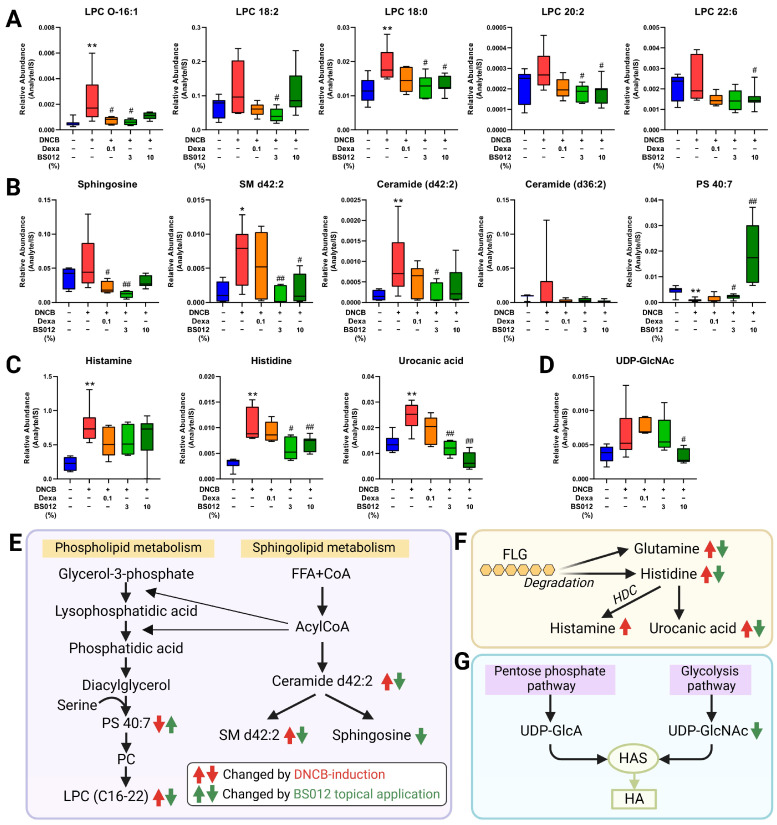
Effect of topical application of BS012 on skin tissue metabolites related to skin barrier function in DNCB-induced mice. The altered levels of identified metabolites related to (**A**) Glycerophospholipid, (**B**) sphingolipid, (**C**) histidine, and (**D**) hyaluronic acid (HA) metabolism showed significant changes in skin tissue. Changes in metabolic pathways related to (**E**) lipid, (**F**) histidine, and (**G**) HA. Graphs depict mean ± SEM, *n* = 6; * *p* < 0.05, ** *p* < 0.01 vs. control group; # *p* < 0.05, ## *p* < 0.01 vs. DNCB group; Dexa: Dexamethasone.

To explore the metabolic alterations in AD-like skin lesions, we analyzed the metabolome of skin tissue, focusing on lipidomics due to the critical role of lipids in forming epidermal lamellae. The metabolic profile of DNCB-induced skin lesions was altered compared to that of the control group. Treatment with dexamethasone and BS012 resulted in distinct clustering (Figure S5). Significant alterations in the 43 metabolites influence various metabolic pathways, including lipid, glutathione (GSH), histidine, amino acid, and polyamine metabolisms (Table S2). These changes are related to improvements in the metabolic abnormalities associated with inflammation, oxidative stress, and skin barrier function in AD. First, changes in FA metabolism related to immunity were identified in skin tissue (Figure 4A), with the altered metabolites mainly related to n-3 and n-6 PUFAs. These changes indicated an improved inflammatory response by BS012 treatment (Figure 4B). Moreover, an upregulation of the pathway related to polyamine, glutamine, the GSH pathway, and uric acid was observed in response to AD induction, with noticeable improvement in metabolism following BS012 application (Figure 5A). These pathways are related to immune regulation and oxidative stress (Figure 5B). Significant alterations were also observed in metabolites associated with several lipid metabolisms, including phospholipid and sphingolipid, histidine-related metabolism, and hyaluronic acid (HA) metabolism (Figure 6A). These metabolites play crucial roles in symptoms such as those associated with AD lesions, such as improving skin barrier function and reducing dryness and itching (Figure 6B). Notably, beneficial metabolic changes related to these symptoms were observed following BS012 treatment. Most of the identified metabolites changed in a pattern opposite to the DNCB-induced changes after dexamethasone and BS012 administration, significantly improving the DNCB-induced metabolic imbalance.

### 3.5. Identification of Key Components of BS012 through the Profiling of Exogenous Metabolites in Skin Tissues following Topical Application

Profiling was conducted to identify the key components demonstrating activity absorbed into the skin tissue, which revealed 29 metabolites derived from BS012 (Table S3). Among *C. cassia*-derived compounds, 11 essential oil compounds, 10 flavonoids, and two polyphenols were identified with the most components. The compounds from *P. grandiflorum* were detected as four triterpenoid saponins. Additionally, lignan and *N*-acyl amines, specifically *N*-isobutyl-dedecatetraenamide, were detected in *A. sieboldii*.

## 4. Discussion

### 4.1. The Topical Administration of BS012 Reduced Systemic Inflammation and Improved Metabolic Dysfunctions in the Serum of DNCB-Induced NC/Nga Mice 

To evaluate the systemic metabolic mechanism of BS012 on AD treatment, we initially assessed the reduction in symptoms and inflammatory markers typically observed in AD. AD is generally characterized by a mast cell activation and infiltration due to increased IgE levels and the subsequent overexpression of inflammatory cytokines [13]. Additionally, the Th2 inflammatory response triggered by the upregulation of TSLP—a regulator of Th2 immune—is accompanied by itching [14]. This reaction can cause the skin to become excessively dry or thickened, resulting in a loss of skin barrier function [15,16]. Our findings demonstrate that the topical application of BS012 caused a reduction in serum IgE, Th2-mediated cytokine (IL-4), and TSLP levels. Thus, BS012 effectively restored the disrupted balance of Th1 and Th2 cytokines in DNCB-induced NC/Nga mice by suppressing the Th2 response and IgE levels. 

Consistent with the assessed AD-related markers, metabolic changes in the serum induced by BS012 application were associated with anti-inflammatory activity. Regarding serum metabolism, FA metabolism related to the inflammatory response was significantly altered in the DNCB group. n-6 PUFAs and related lipid mediators are mainly involved in the pathogenesis of multiple chronic inflammatory diseases [17]. LOX mediates the oxidation of PUFAs and generates various bioactive lipid mediators [18]; arachidonic-acid-derived lipid mediators by LOX pathways, HETE, and Leukotriene4 play a role in the recruitment and activation of neutrophils and stimulation of the inflammatory pathway in macrophages [19]. HETE, a lipid mediator produced from arachidonic acid by the LOX enzyme, acts as a chemoattractant that triggers the influx of immune cells into the skin in AD skin lesions, contributing to the histological features observed in AD skin lesions [20,21]. HODE, produced from linoleic acid, is a known biomarker of inflammation and oxidative stress [22]. The decreased levels of n-6 PUFA-related metabolites, such as linoleic acid, HODE, HETE, and HpETE, an intermediate of the LOX pathway, after BS012 application could be linked to the regulation of the pro-inflammatory response induced by DNCB (Figure 3). Additionally, in relation to systemic inflammation, the spleen plays a crucial role in maintaining systemic immune responses, and enlargement of the spleen is indicative of a dysregulated immune response in AD [23]. Therefore, the significantly reduced spleen weight in the BS012-treated group, shown in Figure S1, supports the metabolic changes in serum associated with alleviating systemic inflammation. We observed no significant differences in n-3 PUFAs compared to the effect seen with oral administration of BS012. However, alterations in n-6 PUFAs were similar. This suggests that the metabolic changes responsible for inhibiting pro-inflammatory effects play a significant role in systemic immune regulation, even with topical application.

This collective evidence strongly suggests that the topical application of BS012 demonstrates a remarkable inhibitory impact on key pro-inflammatory allergic markers present in serum, including IgE, IL-4, IL-17, and TSLP. Furthermore, BS012 attenuated the increase in PUFAs and lipid mediator metabolism induced by DNCB treatment. 

### 4.2. BS012 Topical Application Attenuates Immune Dysregulation and Oxidative Stress in DNCB-Induced NC/Nga Mouse Skin Lesions

An increase in inflammatory cell infiltration and dermatitis score in skin lesions induced by DNCB indicated the pathological state of AD lesions. The topical application of BS012 resulted in a significant improvement in inflammatory markers and histopathological analysis of skin lesions (Figure 1 and Figure 2). However, because we could not investigate the metabolic mechanism induced by BS012 in skin tissue from the previous study, we conducted this present study to explore the immune- and symptom-related metabolic mechanisms within skin lesions in vivo. The results revealed a significantly greater impact than what was observed in the in vitro AD model. This allowed us to confirm the influence of various metabolic mechanisms (Figure 4, Figure 5 and Figure 6).

In conjunction with these changes, metabolomic and lipidomic analyses of the skin tissue have revealed metabolic changes associated with immune regulation. Regarding FA metabolism, similar to the serum results, several metabolites related to PUFAs and lipid mediators were changed by BS012 topical applications. Dihydroxyoctadecenoic acid (DiHOME), produced by the LOX pathway from linoleic acid, is a risk factor for AD related to microbial disruption in the skin and is involved in impaired late epidermal differentiation in AD caused by the autocrine induction of inflammation [24]. In our study, a significant decrease in linoleic acid and DiHOME was observed following the topical application of BS012 (Figure 4). In contrast, increased levels of eicosapentaenoic acid, an n-3 PUFA, were observed in the BS012 treatment group, and it plays an important role in resolving the onset of AD, and its reduced levels in the skin of atopic patients have been reported [25]. Considering the changes in mast cell infiltration and PUFA metabolism in skin lesions, the topical application of BS012 exerts anti-inflammatory effects by suppressing immune cell influx and pro-inflammatory metabolism.

Biogenic amines in the polyamine pathway regulate cell proliferation and immunity and are associated with DNA protection against oxidative damage [26]. Previous studies confirmed increased levels of acetylspermidine in keratinocytes after TNF-α/IFN-γ stimulation, and the level of spermidine was additionally changed in the DNCB-induced group in our results [8]. Increased levels of asparagine, a non-essential amino acid, in AD skin lesions compared to in non-lesional skin play an important role in cellular responses to amino acid homeostasis and urea cycle metabolism. Asparagine can be converted to aspartate and act as a metabolic regulator of non-essential amino acids including glutamine and glutamate [27]. Glutamine is an important energetic and biosynthetic nutrient utilized by immune cells such as lymphocytes, macrophages, and neutrophils [28]. Glutamate, an important intermediate in glutamine metabolism, is involved in glutamine biosynthesis and in the synthesis of GSH, an antioxidant compound [29]. Glutamate levels were reported to be more than twice as high in AD lesions compared to in non-lesions and were upregulated in the blood of patients with rheumatoid arthritis, which have a strong inflammatory response [26,30]. Increased levels of spermidine, *N*-acetylspermidine, glutamate, glutamine, and aspartate in AD-like lesions induced by DNCB could be responsible for immune cell modulation and the impaired synthesis of antioxidant compounds; these abnormal metabolic changes were alleviated by the application of BS012 (Figure 5A).

Oxidative stress results from an imbalance between biological antioxidants and reactive oxygen species (ROS). GSH is a powerful endogenous antioxidant that scavenges ROS. Excessive production of ROS leads to the toxic accumulation of oxidized glutathione (GSSG) under pathological conditions, resulting in an imbalance in the redox potential and depletion of GSH [31]. Therefore, the GSH–GSSG ratio is an important marker of oxidative stress [32]. After the topical application of BS012, GSH and GSSG increased and decreased, respectively, and the GSH–GSSG ratio significantly increased in both the 3% and 10% BS012 treatment groups. (Figure 5B). Uric acid is a marker of oxidative stress in chronic skin wounds and is one of the metabolites significantly higher in the dermatitis model than in the wild type [33]. Therefore, these changes in oxidative-stress-related metabolites indirectly implicate the effect of reducing oxidative stress by the topical application of BS012.

### 4.3. Topical Application of BS012 Improved Skin Barrier Function and AD Symptoms in Damaged Skin Lesions of DNCB-Induced NC/Nga Mice 

In AD, the overactivated type 2 immune response blocks the terminal differentiation of keratinocytes and the formation of a mature *stratum corneum*, contributing to the skin barrier function. The skin barrier dysfunction in the lesions of patients with AD weakens the ability to protect against transepidermal water loss (TEWL) and prevents the penetration of allergens and pathogens into the skin, resulting in the worsening of AD symptoms. The lipid composition of *stratum corneum* plays an important role in the skin barrier and differs significantly between patient with AD and healthy subjects [34]. Therefore, the metabolomics and lipidomics were performed on lesional tissues to investigate the effect of BS012 topical treatment on the lipid composition in the *stratum corneum*. 

As shown in Figure 6A, the level of lysophosphatidylcholine (LPC) species with short carbon chains (C < 24) tended to be upregulated in the DNCB-treated group and was significantly downregulated, close to the control level, after BS012 topical treatment. Downregulation of the elongation of long-chain fatty acid family members 3 and 6 (ELOVL3 and ELOVL6) in AD lesions is consistent with the expression of long-chain fatty acid-containing LPC [34]. A shift in lipid molecules toward shorter chains in the AD group was observed not only in LPC but also in sphingolipid metabolism. In the lesions of patients with AD, there is a decrease in the ceramide (CER) species with carbon chains of C50 or larger compared to those in healthy individuals, whereas species with chains of C46 or smaller show an increase [35]. CER d42:2 increased in the affected skin tissue and decreased after BS012 application; similar patterns were observed for SM d42:2 and sphingosine (Figure 6B,E). These changes may be associated with the role of sphingolipid metabolism in maintaining the integrity and permeability of lipid lamellae [36]. The imbalance in lipid composition observed in the AD group has been consistently reported in previous studies using in vitro keratinocyte models [8]. These changes reflect metabolic alterations that impact the dysfunction of the skin barrier function and TEWL. The group with the topical application of BS012 demonstrated a more significant level of improvement in this imbalance than that of the Dexa group. 

Downregulation of phosphatidylserine (PS) due to AD induction increased after applying 10% BS012. PS is well known for its various effects on improving overall skin conditions, including the skin barrier function and suppression of inflammatory responses in atopic-like dermatitis [37]. An increase in PS levels is expected to indirectly contribute to symptom improvement. Therefore, the increased level of PS 40:7 due to BS012 administration is expected to have metabolic effects that contribute to symptom improvement.

Intractable and intense itching is a representative symptom that significantly reduces the quality of life of patients with AD [38]. Constant skin stimulation by itching and scratching can cause chronic inflammatory eczema; therefore, controlling itching is important in treating AD. As shown in Figure S2, the total scratching behavior significantly increased after DNCB treatment. Therefore, we identified metabolic markers that cause itching in AD. The concentration of dermatitis-associated metabolites, such as histamine, urate, and serotonin, in the dermatitis-affected skin of Mdr1a/1b/Bcrp^-/-^ mice was higher than that in wild-type mice [33]. A decrease in uric acid and histamine levels was observed in the BS012 treatment group (Figure 5C and Figure 6C). Histamine is a mediator released from activated mast cells along with pro-inflammatory cytokines and accumulates in the skin of patients with AD, worsening itching [39]. These changes in the metabolic markers of itching support a reduction in scratching behavior. Histamine metabolism is also related to the expression of filaggrin (FLG), a filament-associated protein that plays a crucial role in the terminal differentiation of keratinocytes and maintains skin barrier integrity [40]. Histidine is a precursor that produces histamine via histidine decarboxylase and is a major constituent of FLG [41]. Epidermal barrier defects associated with FLG deficiency play a critical role in AD pathogenesis [42]. In addition, urocanic acid (UCA) is an intermediate in histidine catabolism and cis-UCA induces intracellular ROS, causing oxidative DNA damage [43]. Dysregulation of histidine metabolism by DNCB induction may be associated with an increase in the amino acid constituents, glutamine, and histidine, due to the degradation of FLG induced by DNCB. The related metabolic imbalance was improved by the topical application of BS012 (Figure 6F).

HA is a non-sulfated glycosaminoglycan composed of repeating units of *N*-acetyl-glucosamine (GlcNAc) and glucuronic acid (GlcA), and it is a basic component of the extracellular matrix that contributes to skin hydration, the regulation of inflammation, and wound healing [44]. UDP-GlcNAc and UDP-GlcA were used as substrates for the hyaluronic acid synthesis enzyme (HAS). Therefore, decreased levels of UDP-GlcNAc after BS012 treatment may be associated with increased UDP-GlcNAc availability and enhanced HA synthesis (Figure 6D,G). These results suggest that BS012 may improve skin hydration and barrier function in atopic dermatitis skin lesions.

## 5. Conclusions

In our previous study, we established the positive impact of BS012 on alleviating the symptoms of skin barrier dysfunction associated with AD using an in vitro AD model, suggesting its potential as a topical agent. Additionally, the safety of the topical application of BS012 was evaluated through cytotoxicity evaluation in keratinocytes in a previous study [8]. Therefore, in this study, we evaluated the improvement in AD pathology and symptoms by the topical application of BS012 in vivo and investigated the metabolic mechanisms of the therapeutic effect in the whole body and lesional skin using a metabolomic approach.

**Figure 7:**
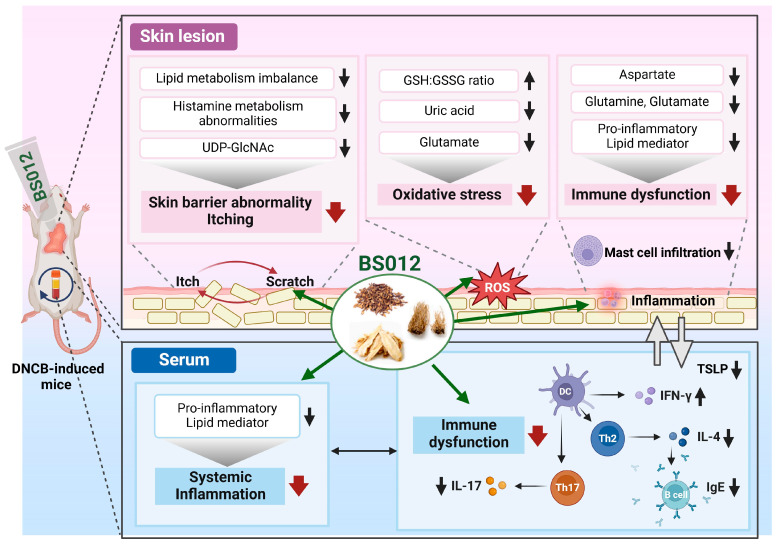
The molecular and metabolic mechanisms of the topical application of BS012 on systemic and lesioned skin in DNCB-induced mice. The black arrows represent the changes in inflammation and metabolic markers reduced by BS012, while the red arrows indicate the decreasing effects of BS012 on pathological changes observed in atopic dermatitis (AD).

In DNCB-treated mice, the topical application of BS012 improved systemic inflammation by suppressing Th2-specific immune responses and modulating inflammation-related FA metabolism. Moreover, within the lesional skin, BS012 improved irregularities and alleviated AD symptoms through alterations in metabolism, leading to an enhancement in the skin barrier function and suppression of oxidative stress (Figure 7). These results revealed that the topical application of BS012 is effective in regulating immunity both locally and systemically. This study, along with our previous study, suggests the potential of BS012 as an oral agent as well as a topical agent for AD treatment and provides crucial insights into the underlying metabolic mechanisms for developing natural therapeutics for AD.

## Data Availability

Data is contained within the article or Supplementary Material.

## Supplementary Materials

The following supporting information can be downloaded at https://www.mdpi.com/article/10.3390/antiox13050563/s1, Figure S1: Relative spleen weight, Figure S2: Effect of BS012 on the scratching behavior in NC/Nga mice, Figure S3: Effect of BS012 on the epidermal thickness in DNCB-induced NC/Nga mice, Figure S4. High-resolution original images of histological analysis. Examinations using H&E and toluidine blue staining at a magnification of 100×, Figure S5: Effects of BS012 topical application on serum metabolome in DNCB-induced mice, Figure S6: Effects of BS012 topical application on skin metabolome in DNCB-induced mice, Table S1: List of identified metabolites significantly altered in serum from DNCB-induced mice with topical BS012 treatment, Table S2: List of identified metabolites significantly altered in skin lesions from DNCB-induced mice with topical BS012 treatment, Table S3: List of the BS012-derived exogenous metabolites detected from skin tissue.
