# Supplementary material for: Exploring the Metabolic Effects of a Herbal Remedy of Asarum sieboldii, Platycodon grandiflorum, and Cinnamomum cassia Extracts: Unraveling Its Therapeutic Potential as a Topical Application for Atopic Dermatitis Treatment
Source: Antioxidants (Basel). 2024 May 2;13(5):563. doi: 10.3390/antiox13050563 (PMC11117881; doi:10.3390/antiox13050563)
Supplement: Supplementary file 1 [file antioxidants-13-00563-s001.zip › antioxidants-2939111-supplementary.pdf]

antioxidants

MDPI

Supplementary Materials

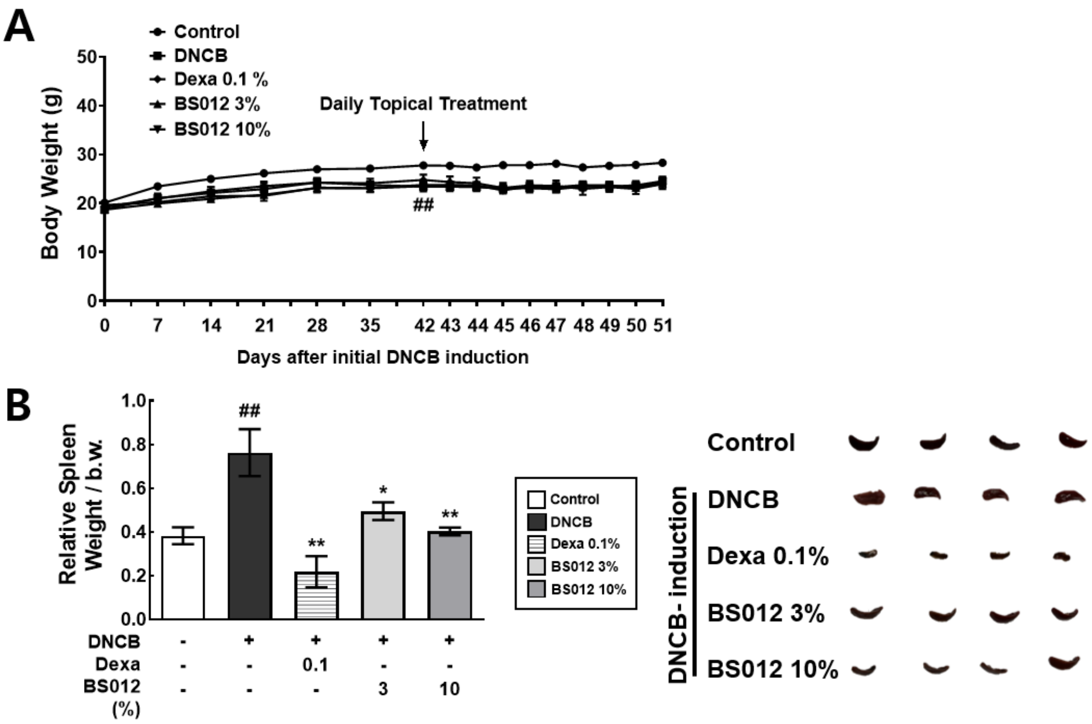

Figure S1. Relative spleen weight. The graphs represent mean ± SEM, n = 6; ##p < 0.01 vs. control group; *p < 0.05, **p < 0.01 vs. DNCB group, Dexa: Dexamethasone.

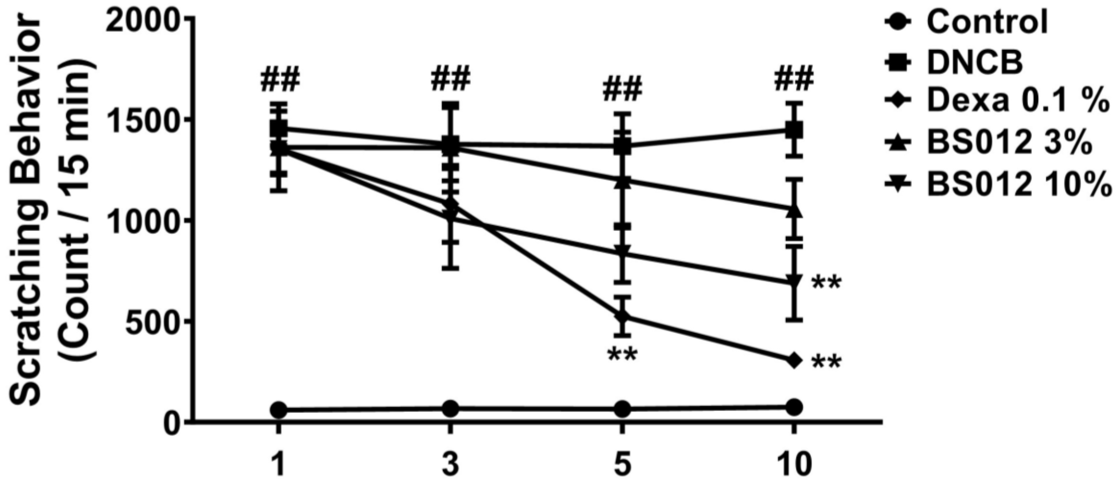

Figure S2. Effect of BS012 on the scratching behavior in NC/Nga mice (A) Total scratching behavior. Scratching behavior was quantified on days 1, 3, 5, and 10 post-treatment. The graphs represent mean ± SEM, n = 6; ##p < 0.01 vs. control group; **p < 0.01 vs. DNCB group, Dexa: Dexamethasone.

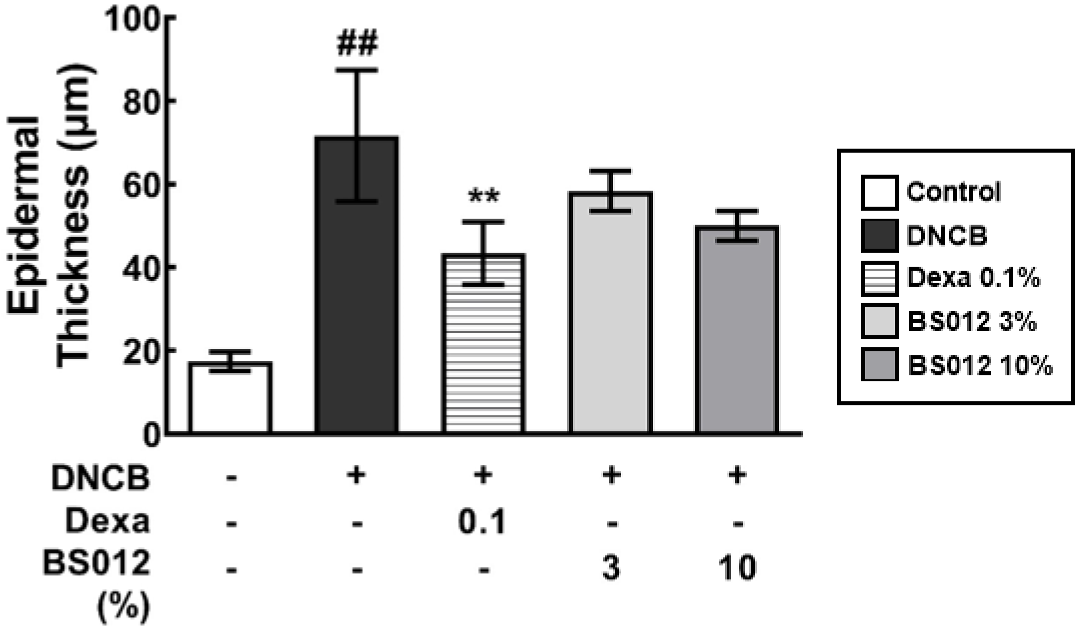

Figure S3. Effect of BS012 on the epidermal thickness in DNCB-induced NC/Nga mice. Epidermal thickness in the skin were evaluated using H&E staining. Data in the graphs are presented as mean ± SEM. ##p < 0.01 vs. control group; **p < 0.01 vs. DNCB-induced group, Dexa: Dexamethasone.

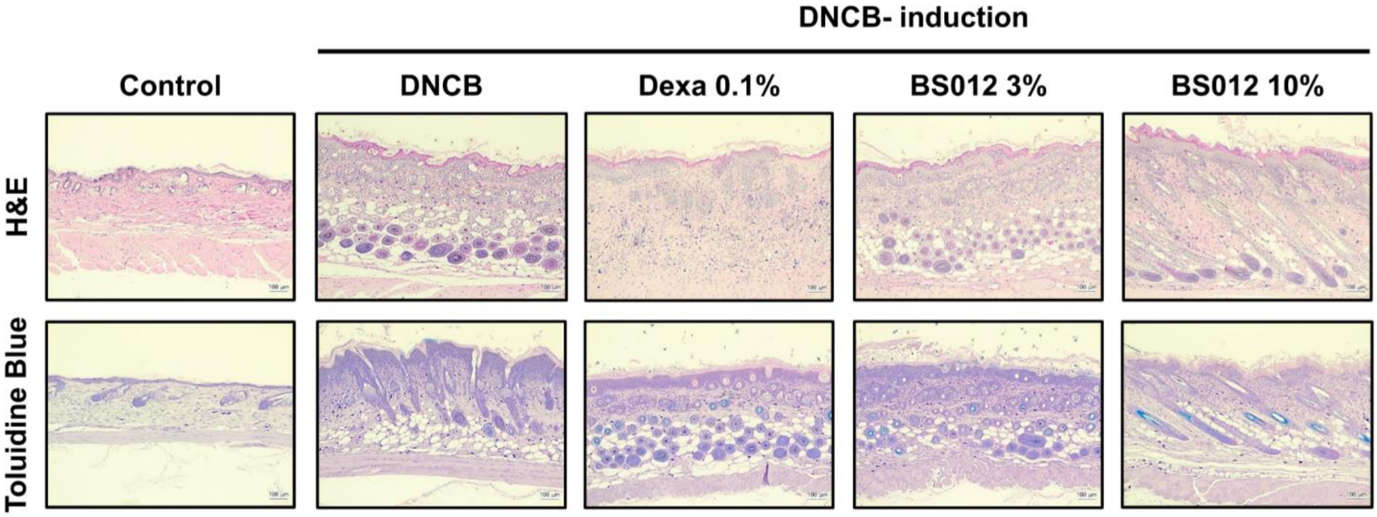

Figure S4. High-resolution original image of histological analysis. Examinations were conducted using H&E and toluidine blue staining at a magnification of 100×.

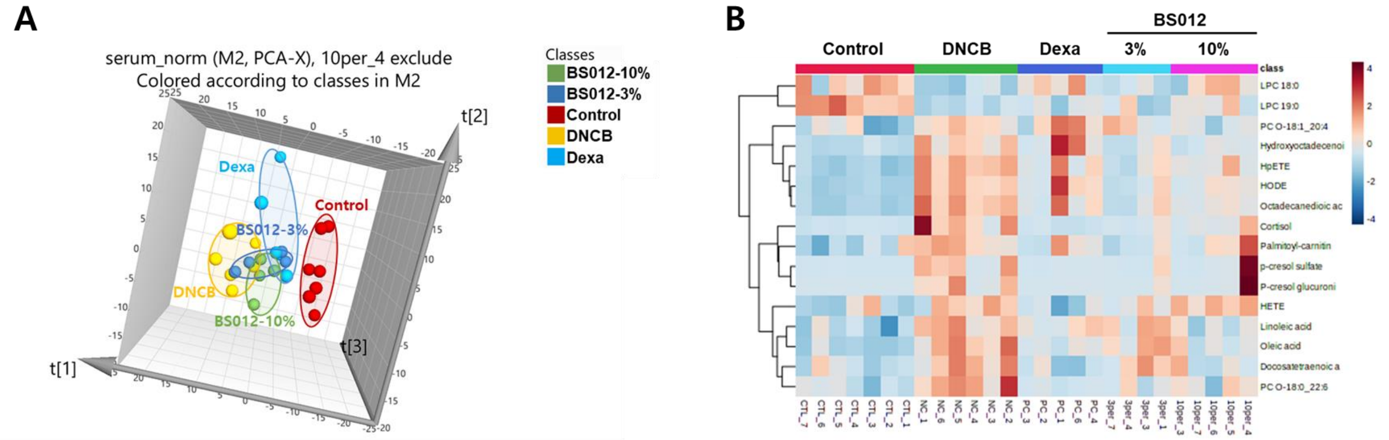

Figure S5. Effects of BS012 topical application on serum metabolome in DNCB-induced mice. (A) The principal component analysis (PCA) score plot derived from serum metabolomics. (B) Heatmap analysis using the 16 identified metabolites. The mean-centered relative abundances divided by the standard deviation of each variable. Dexa: Dexamethasone 0.1%.

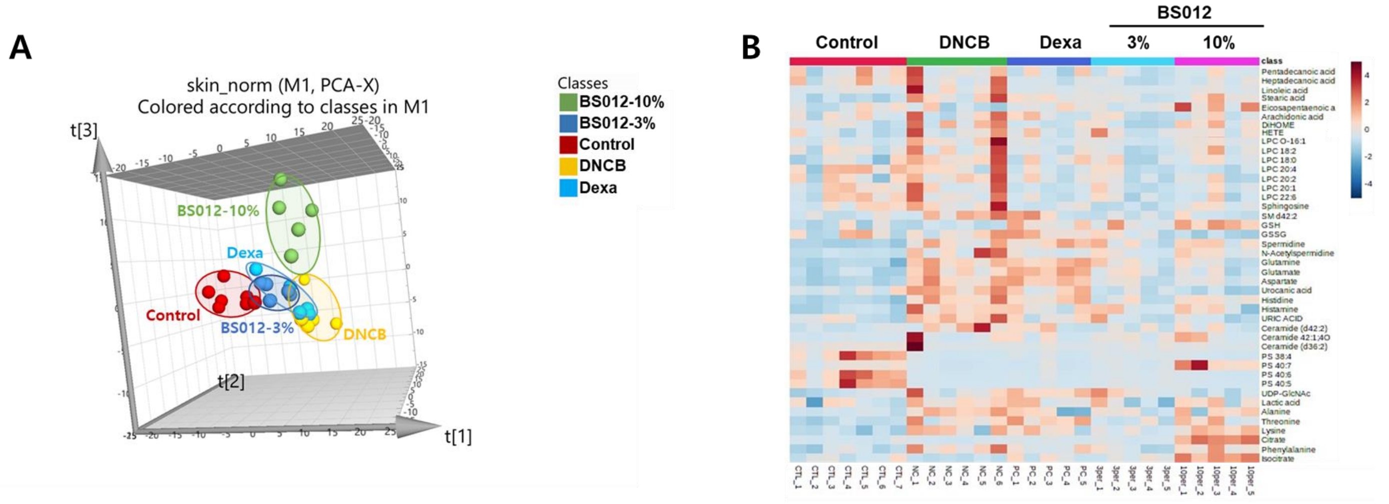

Figure S6. Effects of BS012 topical application on skin metabolome in DNCB-induced mice. (A) The principal component analysis (PCA) score plot derived from serum metabolomics. (B) Heatmap analysis using the 43 identified metabolites. The mean-centered relative abundances divided by the standard deviation of each variable. Dexa: Dexamethasone.

Table S1. List of identified metabolites significantly altered in serum from DNCB-induced mice with topical BS012 treatment.

Related metabolism	Identified metabolite	(ionization mode) Observed m/z	Mean value[a]					Trend[b]	Chage trend[c]		
			Control	DNCB	BS012 (%)		Dexa	Control vs. DNCB[d]	DNCB vs. treated group		
									BS012 (%)		Dexa
					3	10			3	10	
Fatty acid metabolism	Linoleic acid	(-) 279.231	0.183	0.312	0.296	0.224	0.259	↑**	↓	↓*	↓
	Oleic acid	(-) 281.247	0.061	0.174	0.156	0.108	0.100	↑**	↓	↓*	↓**
	HODE	(-) 295.226	0.036	0.224	0.109	0.133	0.197	↑**	↓	↓**	↓
	Hydroxyoctadecenoic acid	(-) 297.241	0.025	0.121	0.057	0.053	0.138	↑**	↓*	↓**	↑
	Octadecanedioic acid	(-) 313.236	0.029	0.163	0.077	0.087	0.114	↑**	↓*	↓**	↓
	HETE	(-) 319.226	2.640	3.251	2.976	3.433	2.520	↑*	↓	↓	↓*
	Docosatetraenoic acid	(-) 331.262	0.023	0.041	0.036	0.027	0.019	↑**	↓	↓	↓**
	HpETE	(-) 381.222	0.004	0.015	0.008	0.010	0.011	↑**	↓**	↓*	↓
Glycero-phospholipid metabolism	LPC 18:0	(-) 568.357	0.279	0.135	0.174	0.246	0.255	↓**	↑	↑	↑*
	LPC 19:0	(-) 582.372	0.084	0.038	0.044	0.056	0.046	↓**	↑	↑*	↑
	PC O-18:1_20:4	(-) 838.592	0.004	0.006	0.006	0.005	0.007	↑*	↓	↓*	↑

	PC O-18:0_22:6	(-) 864.608	0.006	0.010	0.006	0.007	0.006	↑**	↓*	↓	↓**
Others	Palmitoyl-carnitine	(+) 400.342	0.033	0.065	0.039	0.059	0.051	↑**	↓*	↓	↓
	p-Cresol sulfate	(-) 187.006	0.005	0.984	0.249	0.876	0.052	↑**	↓*	↓	↓*
	p-Cresol glucuronide	(+) 302.122	0.000	0.003	0.001	0.003	0.000	↑**	↓*	↑	↓*
	Cortisol	(-) 361.199	0.005	0.073	0.014	0.027	0.004	↑**	↓*	↓	↓**

[a]Average values of relative abundance normalized by internal standard in each group. [b]Change trends in the DNCB group compared to the control group. [c]Change trends in the BS012 or dexamethasone treated group compared to the DNCB group. $^{\#}p < 0.05$, $^{\#\#}p < 0.01$ vs. Control group; $^{*}p < 0.05$, $^{**}p < 0.01$ vs. DNCB group, Dexa: Dexamethasone.

Table S2. List of identified metabolites significantly altered in skin lesions from DNCB -induced mice with topical BS012 treatment.

Related metabolism	Identified metabolite	(ionization mode) Observed m/z	Mean value[a]					Trend[b]	Change trend[c]		
			Control	DNCB	BS012 (%)		Dexa	Control vs. DNCB[d]	DNCB vs. treated group[e]		
									BS012 (%)		Dexa
					3	10			3	10	
Fatty acid metabolism	Pentadecanoic acid	(-) 241.217	0.0180	0.0205	0.0128	0.0164	0.0149	↑	↓*	↓	↓
	Heptadecanoic acid	(-) 269.249	0.0678	0.0810	0.0334	0.0496	0.0392	↑	↓*	↓	↓
	Linoleic acid	(-) 279.232	0.0010	0.0037	0.0008	0.0011	0.0009	↑	↓*	↓	↓
	Stearic acid	(-) 283.263	0.0001	0.0005	0.0003	0.0004	0.0004	↑**	↓*	↓	↓
	Eicosapentaenoic acid	(+) 303.233	0.0086	0.0062	0.0085	0.0195	0.0098	↓	↑	↑**	↑
	Arachidonic acid	(-) 303.232	0.0084	0.0125	0.0068	0.0072	0.0094	↑	↓	↓	↓
	DiHOME	(-) 313.238	0.0028	0.0056	0.0022	0.0051	0.0027	↑	↓*	↓	↓
	HETE	(-) 319.227	0.0002	0.0005	0.0004	0.0003	0.0003	↑	↓	↓	↓
Polyamine metabolism	Spermidine	(+) 147.077	0.0019	0.0056	0.0049	0.0051	0.0060	↑**	↓	↓	↑
	N-Acetylspermidine	(+) 188.175	0.0245	0.3343	0.1219	0.1842	0.1091	↑**	↓	↓	↓
	Glutamine	(-) 145.061	0.0074	0.0191	0.0146	0.0109	0.0199	↑**	↓	↓**	↑
	Glutamate	(-) 146.046	0.0104	0.0177	0.0141	0.0113	0.0215	↑**	↓	↓**	↑
	Aspartate	(-) 132.030	0.0050	0.0129	0.0083	0.0076	0.0135	↑**	↓**	↓**	↑
Glutathione metabolism	GSH	(+) 308.091	0.0045	0.0042	0.0084	0.0133	0.0107	↓	↑*	↑**	↑**
	GSSG	(+) 613.160	0.0055	0.0065	0.0046	0.0019	0.0076	↑	↓	↓**	↑
Glycerophospholipid metabolism	LPC O-16:1	(-) 524.334	0.0006	0.0023	0.0006	0.0011	0.0007	↑**	↓*	↓	↓*
	LPC 18:2	(+) 520.340	0.0692	0.1201	0.0430	0.1135	0.0596	↑	↓*	↓	↓
	LPC 18:0	(-) 568.361	0.0119	0.0191	0.0127	0.0131	0.0147	↑**	↓*	↓*	↓
	LPC 20:4	(+) 544.342	0.0057	0.0059	0.0033	0.0033	0.0036	↑	↓	↓	↓
	LPC 20:2	(-) 592.360	0.0002	0.0003	0.0002	0.0002	0.0002	↑	↓*	↓*	↓
	LPC 20:1	(+) 550.387	0.0020	0.0029	0.0012	0.0015	0.0010	↑	↓	↓	↓*
	LPC 22:6	(-) 612.329	0.0021	0.0024	0.0015	0.0015	0.0015	↑	↓	↓*	↓
Sphingolipid metabolism	Sphingosine	(+) 300.291	0.0345	0.0570	0.0119	0.0300	0.0221	↑	↓**	↓	↓*
	SM d42:2	(-) 857.675	0.0015	0.0070	0.0009	0.0018	0.0053	↑*	↓**	↓*	↓
	Ceramide (d42:2)	(-) 692.619	0.0002	0.0009	0.0002	0.0005	0.0005	↑**	↓*	↓	↓
	Ceramide (d42:1)	(+) 684.652	0.0005	0.0021	0.0013	0.0018	0.0010	↑	↓	↓	↓
	Ceramide (d36:2)	(-) 622.541	0.0087	0.0208	0.0036	0.0022	0.0022	↑	↓	↓	↓
	PS 38:4	(-) 810.531	0.0486	0.0020	0.0077	0.0025	0.0040	↓**	↑**	↑	↑
	PS 40:7	(-) 832.515	0.0045	0.0009	0.0022	0.0179	0.0013	↓**	↑*	↑**	↑
	PS 40:6	(+) 836.544	0.0282	0.0007	0.0031	0.0011	0.0019	↓**	↑**	↑	↑
	PS 40:5	(-) 836.546	0.0248	0.0008	0.0033	0.0010	0.0014	↓**	↑**	↑	↑
Histidine metabolism	Urocanic acid	(-) 137.035	0.0137	0.0246	0.0119	0.0071	0.0190	↑**	↓**	↓**	↓
	Histidine	(-) 154.062	0.0030	0.0105	0.0058	0.0069	0.0092	↑**	↓*	↓**	↓
	Histamine	(+) 112.087	0.2223	0.7793	0.5591	0.6005	0.5457	↑**	↓	↓	↓
Amino acid metabolism	Alanine	(+) 90.055	0.0292	0.0594	0.0389	0.0468	0.0318	↑**	↓**	↓	↓
	Threonine	(+) 118.051	0.0470	0.1111	0.0665	0.1103	0.1391	↑**	↓	↓	↑
	Lysine	(+) 147.113	0.0064	0.0189	0.0126	0.0195	0.0161	↑*	↓	↑	↓
	Phenylalanine	(+) 120.080	0.1127	0.2429	0.1383	0.2071	0.1753	↑**	↓**	↓	↓
Others	Isocitrate	(-) 173.009	0.0433	0.0194	0.0548	0.2147	0.0562	↓	↑ **	↑**	↑*
	Citrate	(+) 191.019	0.0194	0.0183	0.0265	0.2301	0.0251	↓	↑	↑**	↑
	UDP-GlcNAc	(-) 606.074	0.0037	0.0066	0.0065	0.0034	0.0077	↑	↓	↓*	↑
	Uric acid	(-) 167.021	0.0102	0.0212	0.0115	0.0075	0.0133	↑**	↓*	↓**	↓
	Lactic acid	(-) 89.0244	0.2902	0.3570	0.2752	0.3487	0.3490	↑	↓*	↓	↓

[a]Average values of relative abundance normalized by internal standard in each group. [b]Change trends in the DNCB group compared to the control group. [c]Change trends in the BS012 or dexamethasone treated group compared to the DNCB group. $^{\#}p < 0.05$, $^{\#\#}p < 0.01$ vs. Control group; $^{*}p < 0.05$, $^{**}p < 0.01$ vs. DNCB group, Dexa: Dexamethasone.

Table S3. List of the BS012-derived exogenous metabolites detected from skin tissue.

	Classification	Compounds	Retention time (min)	Molecular formula	Measured m/z	Adduct	MS/MS fragment m/z (Relative intensity, %)
Cinnamomum cassia	Essential oil components	Cinnamaldehyde	10.50	C_9H_8O	115.0543	$[M+H-H_2O]^+$	116 (100), 107 (80), 88 (75)
		Methoxycinnamaldehyde	10.06	$C_{10}H_{10}O_2$	163.0755	$[M+H]^+$	135 (100), 145 (80), 107 (60)
		Hydroxycinnamic acid	7.94	$C_9H_8O_3$	147.0441	$[M-H]^-$	119 (100)
		Hydroxymethoxycinnamate	7.30	$C_{10}H_{10}O_4$	177.0549	$[M+H-H_2O]^+$	177 (100)
		Dimethoxycinnamic acid	9.28	$C_{11}H_{12}O_4$	209.0813	$[M+H]^+$	191 (100), 161 (20)
		Coumaraldehyde	6.98	$C_9H_8O_2$	149.0598	$[M+H]^+$	131 (100), 121 (100), 107 (40)
		Coumaric acid	7.94	$C_9H_8O_3$	163.0396	$[M-H]^-$	119 (100)
		Methylcoumarin	8.96	$C_{10}H_{10}O_3$	161.0598	$[M+H-H_2O]^+$	161 (100)
		Coumaroyl Hexoside	7.18	$C_{15}H_{18}O_8$	325.0919	$[M-H]^-$	163 (100), 119 (20)
		Dihydrocoumaroyl Hexoside	6.98	$C_{15}H_{20}O_8$	327.1078	$[M-H]^-$	165 (100)
		Feruloyltyramine	8.21	$C_{18}H_{19}NO_4$	312.1231	$[M-H]^-$	297 (100), 178 (90), 135 (40), 313 (30)
	Flavonoids	Kaempferol	9.08	$C_{15}H_{10}O_6$	285.0393	$[M-H]^-$	285 (100)
		Isokaempferide	8.07	$C_{16}H_{12}O_6$	299.0915	$[M-H]^-$	255 (100), 271 (70), 284 (20)
		Dihydrokaempferol	7.70	$C_{15}H_{12}O_6$	287.0552	$[M-H]^-$	259 (100), 243 (15)
		Kaempferol-3-O-glucoside-6''-*p*-coumaroyl	8.69	$C_{30}H_{26}O_{13}$	593.1278	$[M-H]^-$	285 (100), 447 (10)
		Quercetin	7.98	$C_{15}H_{10}O_7$	303.0506	$[M+H]^+$	285 (100), 257 (80), 229 (40)
		Dihydroquercetin	7.19	$C_{15}H_{12}O_7$	303.0499	$[M-H]^-$	285 (100), 177 (5), 125 (5)
		Quercetin-3-O-pentoside	7.83	$C_{20}H_{18}O_{11}$	433.0757	$[M-H]^-$	373 (100), 403 (50), 300 (30)
		Quercetin-3-O-rhamnoside	8.03	$C_{21}H_{20}O_{11}$	447.0916	$[M-H]^-$	301 (100)
		Quercetin-4'-O-glucoside	7.62	$C_{21}H_{20}O_{12}$	463.0865	$[M-H]^-$	301 (100)
		Quercetin-3-O-deoxyhexosyl(1-2) pentoside	7.98	$C_{26}H_{28}O_{15}$	579.1344	$[M-H]^-$	300 (100)
	Polyphenols	Syringaldehyde	7.00	$C_9H_{10}O_4$	183.0654	$[M+H]^+$	155 (100), 123 (20)
		Catechin	6.57	$C_{15}H_{14}O_6$	289.0711	$[M-H]^-$	245 (100), 205 (30)
Platycodon grandiflorum	Triterpenoid saponins	Platycodin D	9.42	$C_{57}H_{92}O_{28}$	1223.5704	$[M-H]^-$	1224 (100)
		Polygalacin D	9.50	$C_{57}H_{92}O_{27}$	1207.5752	$[M-H]^-$	665 (100), 469 (65), 1117 (25)
		Platyconic acid D	9.44	$C_{54}H_{84}O_{26}$	1147.5162	$[M-H]^-$	1117 (100), 937 (85), 485 (20)
		Platycosaponin A	8.81	$C_{42}H_{68}O_{16}$	827.4433	$[M-H]^-$	828 (100), 665 (30)
Asarum sieboldii	Lignan	Sesamin	10.65	$C_{20}H_{18}O_6$	337.1080	$[M+H]^+$	319 (100), 267 (45), 289 (35), 135 (20)
	N-acyl amines	*N*-isobutyl-dodecatetraenamide	11.34	$C_{16}H_{25}NO$	248.2019	$[M+H]^+$	149 (100), 142 (60)
